# Probing the interaction between the encapsulated water molecule and the fullerene cages in H_2_O@C_60_^–^ and H_2_O@C_59_N^–^

**DOI:** 10.1039/c8sc01031e

**Published:** 2018-06-04

**Authors:** Guo-Zhu Zhu, Yuan Liu, Yoshifumi Hashikawa, Qian-Fan Zhang, Yasujiro Murata, Lai-Sheng Wang

**Affiliations:** a Department of Chemistry , Brown University , Providence , Rhode Island 02912 , USA . Email: Lai-Sheng_Wang@brown.edu; b Institute for Chemical Research , Kyoto University , Uji , Kyoto 611-0011 , Japan

## Abstract

We report a high-resolution photoelectron imaging study of cryogenically-cooled H_2_O@C_60_^–^ and H_2_O@C_59_N^–^ endohedral fullerene anions. The electron affinity (EA) of H_2_O@C_60_ is measured to be 2.6923 ± 0.0008 eV, which is 0.0088 eV higher than the EA of C_60_, while the EA of H_2_O@C_59_N is measured to be 3.0058 eV ± 0.0007 eV, which is 0.0092 eV lower than the EA of C_59_N. The opposite shifts are found to be due to the different electrostatic interactions between the encapsulated water molecule and the fullerene cages in the two systems. There is a net coulombic attraction between the guest and host in H_2_O@C_60_^–^, but a repulsive interaction in H_2_O@C_59_N^–^. We have also observed low-frequency features in the photoelectron spectra tentatively attributed to the hindered rotational excitations of the encapsulated H_2_O molecule, providing further insights into the guest–host interactions in H_2_O@C_60_^–^ and H_2_O@C_59_N^–^.

## Abstract 2

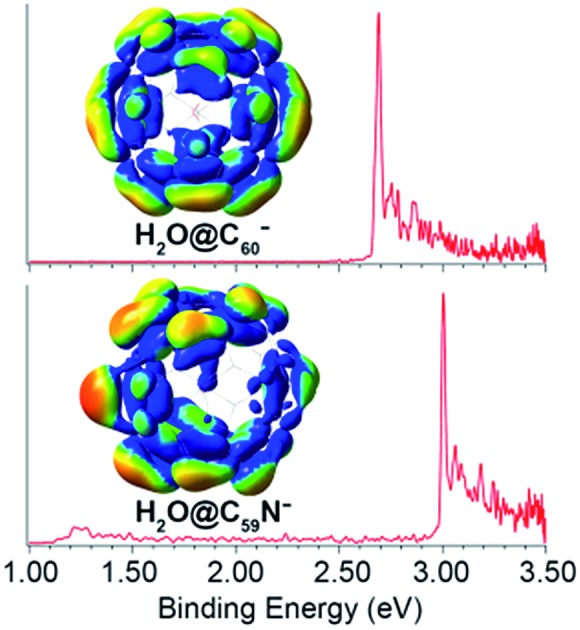
The guest–host interactions in the H_2_O@C_60_ and H_2_O@C_59_N endohedral fullerenes are probed by high-resolution photoelectron imaging.

## 1. Introduction

Endohedral fullerenes with encapsulated atoms, molecules or clusters have attracted wide interest due to their unique electronic, magnetic, and optical properties.^1^–^4^ Since the first observation of the endohedral fullerene La@C_60_ in a mass spectrum in 1985,^5^ a variety of such novel guest–host complexes containing noble gas atoms,^6^,^7^ the N atom and the N_2_ molecule,^8^ metal atoms and metal clusters,^4^,^9^ have been synthesized using the arc discharge or ion bombardment methods. These harsh production conditions were unable to make endofullerenes containing light molecules.^10^ A more rational synthetic approach, called molecular surgery on the fullerene surfaces,^11^,^12^ was successfully applied to the macroscopic synthesis of H_2_@C_60_,^13^ followed by the syntheses of H_2_O@C_60_,^14^,^15^ H_2_O@C_59_N,^16^ HF@C_60_,^17^ (H_2_O)_2_@C_70_,^18^ and very recently even (H_2_O·HF)@C_70_.^19^ The H_2_O@C_60_ and H_2_O@C_59_N species are of special interest because the water molecule is isolated without hydrogen bonds. Many experimental and theoretical studies have been carried out to elucidate the novel properties of H_2_O@C_60_, such as its polarity,^20^–^26^ quantum dynamics,^27^–^30^ magnetic,^16^,^31^ mechanical,^32^ thermal^33^ and electric properties^25^,^26^,^34^,^35^ as well as its chemical reactivity.^24^,^36^,^37^

One of the most interesting questions about H_2_O@C_60_ concerned the nature of the guest–host interactions of the water molecule trapped in the C_60_ cage. No detectable difference was observed between the UV-Vis absorption spectra of the empty C_60_ and H_2_O@C_60_, suggesting that the water molecule has very weak interactions with the cage.^14^ This observation was further confirmed by studies of nuclear spin relaxation^31^ and electric conductance.^35^ However, theoretical calculations found strong dispersion interactions^22^,^23^,^38^–^40^ between the free rotating water molecule and C_60_.^14^,^23^,^27^,^40^,^41^ The quantized rotational levels and the nuclear spin-isomerism of *ortho*- and *para*-water in H_2_O@C_60_ were studied by inelastic neutron scattering, far-infrared spectroscopy, and nuclear magnetic resonance.^27^–^29^ These studies revealed a splitting of the ground rotational state of *ortho*-H_2_O and a symmetry-breaking of the C_60_ cage, indicating a quadrupolar interaction between H_2_O and C_60_.^30^ In addition, the dipole moment of H_2_O@C_60_ was measured to be around 0.5 D,^25^,^26^ in good agreement with theoretical calculations.^20^–^24^ The significant reduction of the dipole moment of the encapsulated H_2_O is a result of the strong shielding effect by the nonpolar C_60_ cage. A recent study reported that the rotation of the encapsulated water can be electrostatically perturbed by introducing polarized C(C_60_)–X (X: heteroatom) bonds.^42^

Unlike the extensive studies on H_2_O@C_60_, the H_2_O@C_59_N endohedral azafullerene was only synthesized very recently in the dimer form, (H_2_O@C_59_N)_2_.^16^ The presence of the N atom breaks the symmetry of the fullerene and introduces a polar center. Theoretical calculations suggested an attractive electrostatic interaction between the O atom of H_2_O and the N atom of C_59_N.^16^,^43^,^44^ Comparison of the different guest–host interactions in H_2_O@C_60_ and H_2_O@C_59_N would be very interesting. In particular, the electron affinity (EA) of the endohedral fullerenes can be a good probe of these guest–host interactions, because the extra electron in the C_60_^–^ and C_59_N^–^ anions is expected to be sensitive to the encapsulated H_2_O molecule.

Here, we present a high-resolution photoelectron (PE) imaging study of the H_2_O@C_60_^–^ and H_2_O@C_59_N^–^ anions cooled in a cryogenic ion trap. The EA of H_2_O@C_60_ is accurately measured to be 2.6923 ± 0.0008 eV, which is 0.0088 eV higher than the EA of C_60_,^45^ while the EA of H_2_O@C_59_N is measured to be 3.0058 eV ± 0.0007 eV, which is 0.0092 eV lower than the EA of C_59_N.^46^ The opposite shifts suggest different guest–host interactions between the encapsulated water molecule and the fullerene cages, which are understood by an electrostatic model. A net coulombic attraction between the water molecule and the HOMO electron in H_2_O@C_60_^–^ is found to stabilize the anion and enhance the EA of H_2_O@C_60_ compared to C_60_, while a repulsive interaction in H_2_O@C_59_N^–^ destabilizes the anion and decreases the EA of H_2_O@C_59_N relative to C_59_N. In addition, low-frequency features in the PE spectra are observed and tentatively attributed to the hindered rotational excitations^30^ of the encapsulated H_2_O molecule, providing further insights into the guest–host interactions in H_2_O@C_60_^–^ and H_2_O@C_59_N^–^.

## 2. Experimental method

The experiment was carried out using our third-generation electrospray PE imaging apparatus,^47^ equipped with a cryogenically-cooled Paul trap^48^ and a high-resolution PE imaging lens.^49^ The electrospray solutions were prepared by dissolving H_2_O@C_60_ or (H_2_O@C_59_N)_2_ samples in a mixed solvent of *o*-dichlorobenzene/CH_3_CN (1/3 ratio in volume), to which tetrakis(dimethylamino)ethylene^50^,^51^ was added as a reducing agent. Anions from the electrospray source were guided into a cryogenically-controlled Paul trap operated at 4.5 K and thermally cooled *via* collisions with 1 mTorr He/H_2_ (4/1 in volume) background gas.^48^ The cold anions were pulsed out of the ion trap at a 10 Hz repetition rate into the extraction zone of a time-of-flight mass spectrometer. The desired anions, H_2_O@C_60_^–^ or H_2_O@C_59_N^–^, were selected by a mass gate and photodetached by the third harmonic of a Nd:YAG laser (354.7 nm) and a tunable dye laser in the interaction zone of the imaging lens.^49^ The PE images were inverse-Abel transformed and reconstructed using both pBasex and BASEX.^52^,^53^ The PE spectra were calibrated with the known spectra of Au^–^ at different photon energies. The kinetic energy (KE) resolution was 3.8 cm^–1^ for electrons with 55 cm^–1^ KE and about 1.5% (ΔKE/KE) for KE above 1 eV in the current experiment.

## 3. Results and discussions

### 3.1. The photoelectron images and spectra of H_2_O@C_60_^–^ and H_2_O@C_59_N^–^ at 354.7 nm

**Fig. 1:**
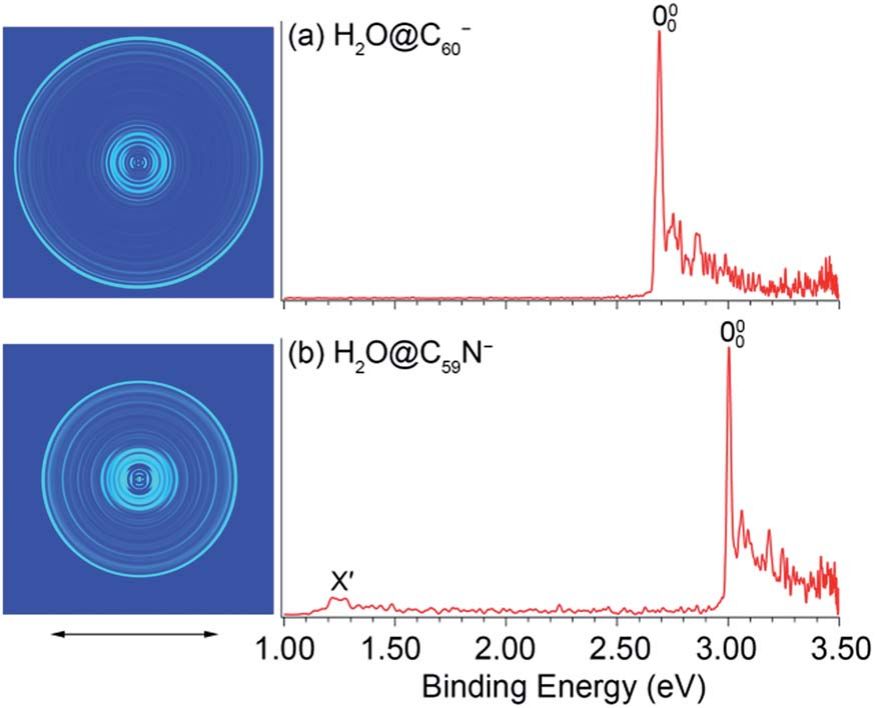
Photoelectron images and spectra of (a) H_2_O@C_60_^–^ and (b) H_2_O@C_59_N^–^ at 354.7 nm. The double arrow below the images indicates the direction of the laser polarization. Note the image corresponding to peak X′ in (b) is cut off.

Fig. 1 shows the PE images and spectra of H_2_O@C_60_^–^ and H_2_O@C_59_N^–^ at 354.7 nm. The first intense peak in each spectrum, labeled as 000, represents the 0–0 transition from the anion to the neutral and defines the EAs for H_2_O@C_60_ and H_2_O@C_59_N, which are measured more accurately in the low photon energy spectra (*vide infra*). The peaks at higher binding energies represent transitions from the ground vibrational state of the anion to the excited vibrational levels of the neutral ground electronic state. They are better resolved in the high-resolution PE images at lower photon energies near the detachment threshold to be discussed below. Fig. 1b also shows a weak peak (X′) at ∼1.2 eV, which is derived from the parent dimer dianion, (H_2_O@C_59_N)_2_^2–^ with the same *m*/*z* as the monoanion. A similar dimer dianion was also observed in the 354.7 nm PE spectrum of C_59_N^–^ recently.^46^ The low binding energy for the dianion was due to the strong intramolecular Coulomb repulsion.^47^,^54^,^55^

**Fig. 2:**
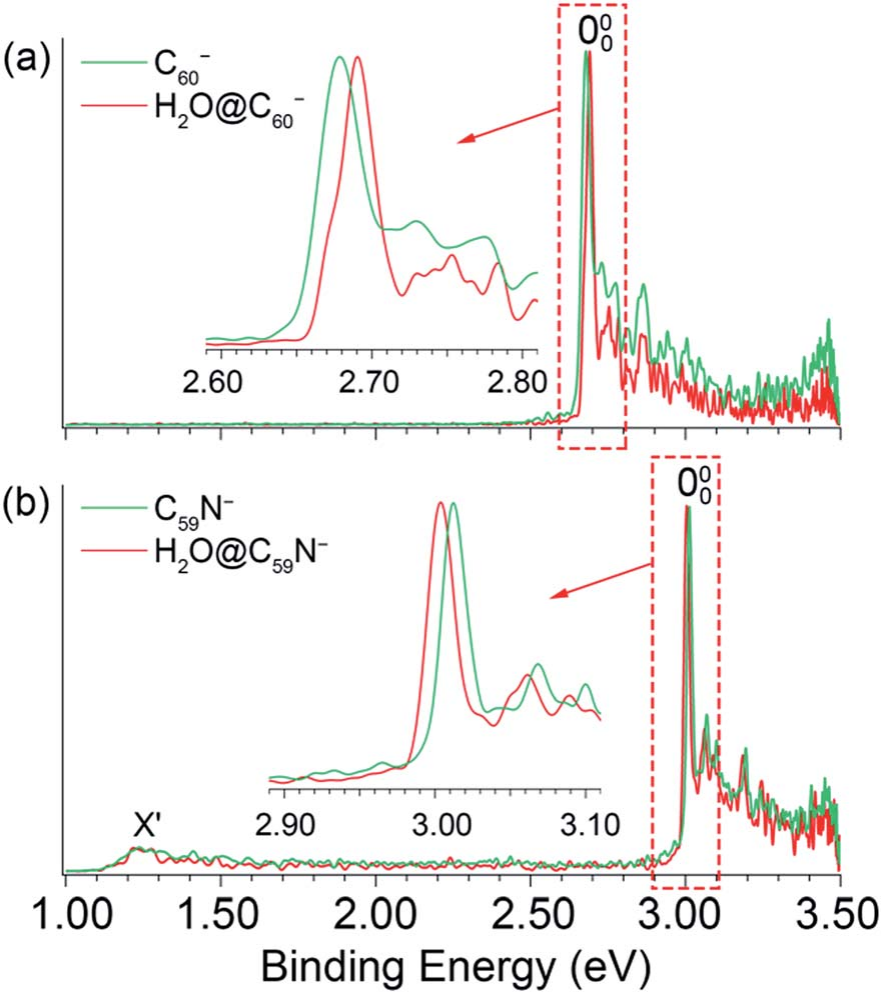
Comparisons of the photoelectron spectra of (a) C_60_^–^ and H_2_O@C_60_^–^, (b) C_59_N^–^ and H_2_O@C_59_N^–^ at 354.7 nm.

The 354.7 nm spectra of the endohedral fullerenes appear to be nearly identical to those of their corresponding parent fullerenes,^45^,^46^ as directly compared in Fig. 2. This observation suggests that indeed the encapsulated water molecule has little effect on the electronic and geometrical structures of the fullerene hosts. However, upon closer examination, a small spectral shift was revealed in each case, as shown in the expanded threshold region given in the respective inset of Fig. 2. Surprisingly, the two endohedral fullerenes exhibit opposite shifts. The electron binding energy of H_2_O@C_60_^–^ was observed to be shifted slightly higher relative to that of C_60_^–^ (Fig. 2a), whereas the electron binding energy of H_2_O@C_59_N^–^ was shifted slightly lower relative to that of C_59_N^–^. The opposite spectral shifts suggest subtle differences in the guest–host interactions of the encapsulated water molecule with the fullerene or azafullerene cages.

### 3.2. The high-resolution photoelectron images and spectra of H_2_O@C_60_^–^ and H_2_O@C_59_N^–^ near detachment thresholds

**Fig. 3:**
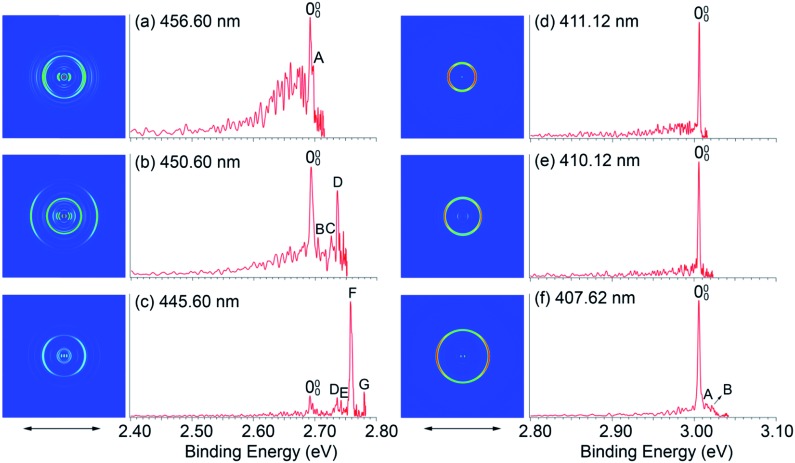
Photoelectron images and spectra of H_2_O@C_60_^–^ at (a) 456.60 nm, (b) 450.60 nm, (c) 445.60 nm and H_2_O@C_59_N^–^ at (d) 411.12 nm, (e) 410.12 nm, (f) 407.62 nm. The double arrows below the images indicate the direction of the laser polarization.

To measure the EAs more accurately and to resolve low-frequency vibrations, we measured PE images for H_2_O@C_60_^–^ and H_2_O@C_59_N^–^ at lower photon energies near the detachment thresholds, as shown in Fig. 3. We found that the detachment cross sections for the endohedral fullerenes were weaker than those of the corresponding empty fullerenes,^45^,^46^ in particular near the detachment thresholds. The spectra shown in Fig. 3 were averaged from 300 000 to 500 000 laser shots. At 456.60 nm (Fig. 3a), the 000 peak with a linewidth of 38 cm^–1^ at an electron kinetic energy of 186 cm^–1^ defines the most accurate value for the EA of H_2_O@C_60_ as 2.6923 ± 0.0008 eV, which is 0.0088 eV higher than the EA of C_60_.^45^ The detachment cross section at this wavelength for H_2_O@C_60_^–^ was particularly poor. The features below the 000 peak in Fig. 3a were partly due to background noise and partly due to hot band transitions, which were amplified relative to the 000 transition. At 411.12 nm (Fig. 3d), the 000 peak with an electron kinetic energy of 80 cm^–1^ and linewidth of 15 cm^–1^ yields the most accurate EA for H_2_O@C_59_N to be 3.0058 eV ± 0.0007 eV, which is 0.0092 eV lower than the EA of C_59_N.^46^

In addition to the near-threshold spectra, two more spectra were taken to resolve low-frequency vibrational features for H_2_O@C_60_ and H_2_O@C_59_N, as shown in Fig. 3b, c, e and f, respectively. There are two types of vibrations for the endohedral fullerenes, one involving the fullerene cages and the other involving the encapsulated water molecules including the hindered rotations. The latter should be particularly sensitive to the guest–host interactions in the endohedral fullerenes. Fig. 3a–c resolve seven vibrational peaks, labeled as A–G for H_2_O@C_60_, while Fig. 3f resolves two peaks, A and B for H_2_O@C_59_N. The relative intensities of the low frequency peaks (A, B) are quite weak for both species, but they seem to be reproducible. The binding energies and shifts relative to the 000 peak for all the vibrational features are summarized in Table 1.

**Table 1:** The observed vibrational peaks, their binding energies (BE) for H_2_O@C_60_^–^ and H_2_O@C_59_N^–^ from the photoelectron spectra in Fig. 3. Their shifts to peak 000 are compared with the vibrational frequencies of C_60_

Anions	Peaks	BE^*a*^ (eV)	Shifts (cm^–1^)	Vib. freq.^*a*^ (cm^–1^)
C_60_^–^		2.6835(6)^*a*^		
H_2_O@C_60_^–^	000	2.6923(8)	0	
	A	2.6967(7)	35	
	B	2.7041(10)	95	
	C	2.7259(10)	271	262
	D	2.7361(7)	353	348
	E	2.7427(10)	406	
	F	2.7582(7)	531	531
	G	2.7803(10)	710	717
C_59_N^–^		3.0150(7)^*b*^		
H_2_O@C_59_N^–^	000	3.0058(7)	0	
	A	3.0151(12)	74	
	B	3.0217(12)	128	

^*a*^ Ref. 45.

^*b*^ Ref. 46.

Peaks C, D, F, G with shifts of 271, 353, 531, and 710 cm^–1^, are similar to those observed in the PE spectra of C_60_^–^ and they should correspond to vibrational modes involving the C_60_ cage.^45^ The strong and highly non-Franck–Condon peak F observed in the 445.60 nm spectrum (Fig. 3c) is also observed for C_60_^–^, which was attributed to strong Hertzberg–Teller coupling.^45^ The weak peak E with a shift of 406 cm^–1^ corresponds to a H_u_(1) vibrational mode of C_60_ also observed by inelastic neutron scattering.^56^ These observations suggest that the H_2_O molecule has little effect on the geometrical and electronic structure of the C_60_ host. Additionally, two weak peaks A and B with small shifts of 35 and 95 cm^–1^, corresponding to very low-frequency transitions, are also tentatively identified. The lowest vibrational frequency of C_60_ is around 260 cm^–1^.^45^,^56^ Hence, these features should correspond to the hindered rotational excitations of the encapsulated water molecule, as revealed by the rigorous full-dimensional quantum calculations of the coupled translation-rotation eigenstates of the water molecule in H_2_O@C_60_.^30^ In Fig. 3f, similarly the two weak peaks A and B with shifts of 74 and 128 cm^–1^ were tentatively identified as the hindered rotational excitations of the encapsulated water molecule. The observation of hindered rotational transitions indicates weak interactions between the encapsulated water molecule and the fullerene cages. The relatively high frequencies observed for the hindered rotational transitions in H_2_O@C_59_N suggest stronger guest–host interactions in this system.

The PE images of H_2_O@C_60_^–^ and H_2_O@C_59_N^–^ in Fig. 3 all exhibit distinct p-wave character with the photoelectron angular distributions parallel to the direction of the laser polarization, similar to those for C_60_^–^ and C_59_N^–^.^45^,^46^ These observations indicate that the encapsulated water molecule does not affect the *s-*like HOMO of the fullerene cages. The p-wave nature of the outgoing electron is partly responsible for the low detachment cross sections near threshold according to the Wigner threshold law.^57^

### 3.3. The opposite shifts of the EAs in H_2_O@C_60_ and H_2_O@C_59_N relative to the empty fullerenes: an electrostatic model

**Fig. 4:**
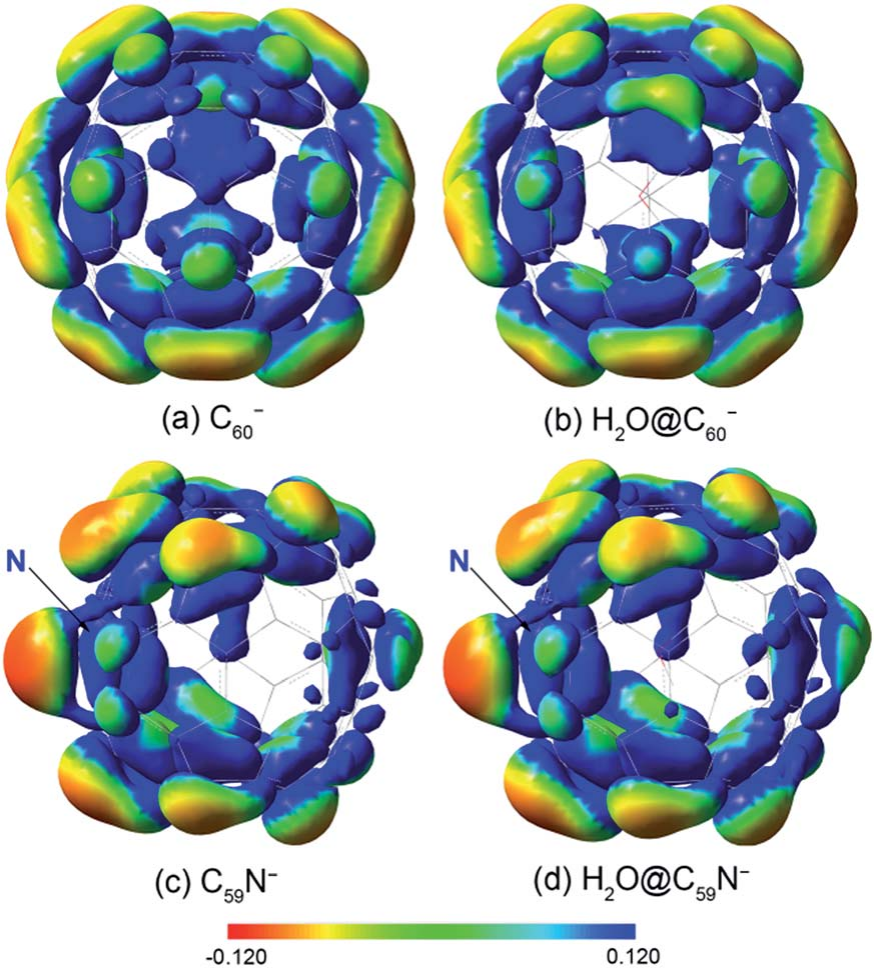
The electrostatic potential maps for the HOMO of (a) C_60_^–^, (b) H_2_O@C_60_^–^, (c) C_59_N^–^, (d) H_2_O@C_59_N^–^, calculated at B3LYP/6-311++G(d,p) level using the GAUSSIAN 09 package.^58^

The opposite shifts of the EAs of H_2_O@C_60_ and H_2_O@C_59_N relative to their corresponding empty cages are consistent with previous theoretical calculations.^24^,^43^ The different effects of the encapsulated water on the EAs can be glimpsed from the electrostatic potential maps of the HOMO of the fullerene anions, as presented in Fig. 4. The extra charge in the half-filled HOMO of C_60_^–^ is evenly distributed on the surface (Fig. 4a). Even though the encapsulated water molecule was known to have no preferred directions,^14^,^23^,^27^,^40^,^41^ it breaks the symmetry and dynamically induces a slightly higher charge density on the cage surface, where the H atoms point to (Fig. 4b). On the contrary, the HOMO of C_59_N^–^ is partially localized on the N atom and the C atoms around the N atom (Fig. 4c).^46^ The water molecule in H_2_O@C_59_N^–^ has been shown to adopt a global minimal structure with the O atom pointing to the N atom of the cage due to a weak N···O attractive interaction.^43^,^44^ Despite its orientation preference, the water encapsulation has relatively little effect on the HOMO of C_59_N^–^ (Fig. 4d). However, this orientation of the water molecule brings the electronegative O atom closer to the extra charge, inducing a repulsive interaction.

A simple electrostatic model is used to understand the interactions between the water molecule and the extra charge in the HOMO of the fullerene cages and to obtain insights about the observed different EA shifts in the two systems. In the model, partial charges on the water molecule are represented by point charges with –2*q* located on the oxygen atom and +*q* on each H atom, where *q* is obtained from a Mulliken population analysis of the total wavefunction of the water molecule. The Coulomb interaction can be expressed as:
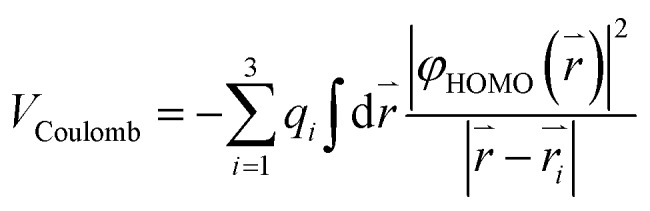
where *q*_*i*_ and *r*_*i*_ represent the charge and position of each atom in the water molecule, *φ*_HOMO_(*r*) is the Kohn–Sham wavefunction of the HOMO of H_2_O@C_60_^–^ and H_2_O@C_59_N^–^ extracted from DFT calculations.^59^ The numerical integration is done with a fine grid converging to 1 meV accuracy. All the geometry optimization and electronic structure calculations were done using DFT at B3LYP/6-311++G(d,p) level of theory with the GAUSSIAN 09 package.^58^

The Coulomb interaction in H_2_O@C_60_^–^ was calculated to be –23 meV, indicating an attractive interaction between the encapsulated water molecule and the HOMO electron in H_2_O@C_60_^–^. This weak attraction, which is in good agreement with previous calculations,^22^,^38^–^40^ stabilizes the H_2_O@C_60_^–^ anion and increases the EA of H_2_O@C_60_ relative to C_60_. On the other hand, the simple electrostatic calculation on the H_2_O@C_59_N^–^ anion yields a repulsive interaction of 64 meV. Hence, the water encapsulation destabilized the HOMO of the C_59_N^–^ anion, reducing the EA of H_2_O@C_59_N relative to C_59_N. This repulsive interaction is expected from the orientation of the H_2_O molecule in C_59_N^–^ and its asymmetric electron density distribution (Fig. 4d). Even though the electrostatic model is rather crude, it correctly predicts the directions of the EA shifts in the two endohedral fullerenes. The interactions between the encapsulated water molecule and the fullerene cages are so weak that they were not detectable in the UV-Vis absorption spectra^14^ or the electrical conductance experiment.^35^

## 4. Conclusions

In conclusion, we report a high-resolution photoelectron imaging study of two endohedral fullerene anions, H_2_O@C_60_^–^ and H_2_O@C_59_N^–^. Accurate electron affinities are obtained for H_2_O@C_60_ (2.6923 ± 0.0008 eV) and H_2_O@C_59_N (3.0058 ± 0.0007 eV) for the first time. The EA of H_2_O@C_60_ is found to be higher than that of C_60_ by 0.0088 eV, whereas the EA of H_2_O@C_59_N is found to be lower than that of C_59_N by 0.0092 eV. These small EA shifts reflect the weak guest–host interactions in the endohedral fullerenes and the opposite shifts are understood using a simple electrostatic model between the encapsulated H_2_O molecule and the HOMO of the fullerene anions. Low-frequency features due to the hindered rotational transitions of the encapsulated water molecule are also tentatively identified, providing further insight into the weak guest–host interactions in the two endohedral fullerenes.

## Conflicts of interest

The authors declare no competing financial interests.

## References

[cit1] Shinohara H. (2000). Rep. Prog. Phys..

[cit2] Guha S., Nakamoto K. (2005). Coord. Chem. Rev..

[cit3] Turro N. J., Chen J. Y. C., Sartori E., Ruzzi M., Marti A., Lawler R., Jockusch S., Lopez-Gejo J., Komatsu K., Murata Y. (2010). Acc. Chem. Res..

[cit4] Popov A. A., Yang S., Dunsch L. (2013). Chem. Rev..

[cit5] Heath J. R., O'Brien S. C., Zhang Q., Liu Y., Curl R. F., Kroto H. W., Tittel F. K., Smalley R. E. (1985). J. Am. Chem. Soc..

[cit6] Weiske T., Bohme D. K., Hrusak J., Kratschmer W., Schwarz H. (1991). Angew. Chem., Int. Ed. Engl..

[cit7] Saunders M., Jimenez–Vazquez H. A., Cross R. J., Poreda R. J. (1993). Science.

[cit8] Murphy T. A., Pawlik T., Weidinger A., Hohne M., Alcala R., Spaeth J.-M. (1996). Phys. Rev. Lett..

[cit9] Wang L. S., Alford J. M., Chai Y., Diener M., Smalley R. E. (1993). J. Phys. D.

[cit10] Levitt M. H. (2013). Philos. Trans. R. Soc., A.

[cit11] Rubin Y. (1997). Chem.–Eur. J..

[cit12] Murata M., Murata Y., Komatsu K. (2008). Chem. Commun..

[cit13] Komatsu K., Murata M., Murata Y. (2005). Science.

[cit14] Kurotobi K., Murata Y. (2011). Science.

[cit15] Krachmalnicoff A., Levitt M. H., Whitby R. J. (2014). Chem. Commun..

[cit16] Hashikawa Y., Murata M., Wakamiya A., Murata Y. (2016). J. Am. Chem. Soc..

[cit17] Krachmalnicoff A., Bounds R., Mamone S., Alom S., Concistrè M., Meier B., Kouřil K., Light M. E., Johnson M. R., Rols S., Horsewill A. J., Shugai A., Nagel U., Rõõm T., Carravetta M., Levitt M. H., Whitby R. J. (2016). Nat. Chem..

[cit18] Zhang R., Murata M., Aharen T., Wakamiya A., Shimoaka T., Hasegawa T., Murata Y. (2016). Nat. Chem..

[cit19] Zhang R., Murata M., Wakamiya A., Shimoaka T., Hasegawa T., Murata Y. (2017). Sci. Adv..

[cit20] Ramachandran C. N., Sathyamurthy N. (2005). Chem. Phys. Lett..

[cit21] Yagi K., Watanabe D. (2009). Int. J. Quantum Chem..

[cit22] Varadwaj A., Varadwaj P. R. (2012). Chem.–Eur. J..

[cit23] Ensing B., Costanzo F., Silvestrelli P. L. (2012). J. Phys. Chem. A.

[cit24] Galano A., Pérez-González A., del Olmo L., Francisco-Marquez M., León-Carmona J. R. (2014). J. Mol. Model..

[cit25] Aoyagi S., Hoshino N., Akutagawa T., Sado Y., Kitaura R., Shinohara H., Sugimoto K., Zhang R., Murata Y. (2014). Chem. Commun..

[cit26] Meier B., Mamone S., Concistre S., Alonso-Valdesueiro J., Krachmalnicoff A., Whitby R. J., Levitt M. H. (2015). Nat. Commun..

[cit27] Beduz C., Carravetta M., Chen J. Y.-C., Concistrè M., Denning M., Frunzi M., Horsewill A. J., Johannessen O. G., Lawler R., Lei X., Levitt M. H., Li Y., Mamone S., Murata Y., Nagel U., Nishida T., Ollivier J., Rols S., Rõõm T., Sarkar R., Turro N. J., Yanga Y. (2012). Proc. Natl. Acad. Sci. U. S. A..

[cit28] Mamone S., Concistrè M., Carignani E., Meier B., Krachmalnicoff A., Johannessen O. G., Lei X., Li Y., Denning M., Carravetta M. (2014). J. Chem. Phys..

[cit29] Goh K. S. K., Jiménez-Ruiz M., Johnson M. R., Rols S., Ollivier J., Denning M. S., Mamone S., Levitt M. H., Lei X., Li Y., Turro N. J., Murata Y., Horsewill A. J. (2014). Phys. Chem. Chem. Phys..

[cit30] Felker P. M., Vlcek V., Hietanen I., FitzGerald S., Neuhauser D., Bacic Z. (2017). Phys. Chem. Chem. Phys..

[cit31] Li Y., Chen J. Y.-C., Lei X., Lawler R. G., Murata Y., Komatsu K., Turro N. J. (2012). J. Phys. Chem. Lett..

[cit32] Min K., Farimani A. B., Aluru N. R. (2013). Appl. Phys. Lett..

[cit33] Gao Y., Xu B. (2015). J. Phys. Chem. C.

[cit34] Zhu C., Wang X. (2015). Sci. Rep..

[cit35] Kaneko S., Hashikawa Y., Fujii S., Murata Y., Kiguchi M. (2017). ChemPhysChem.

[cit36] Maroto E. E., Mateos J., Garcia-Borràs M., Osuna S., Filippone S., Herranz M. Á., Murata Y., Solà M., Martín N. (2015). J. Am. Chem. Soc..

[cit37] Reveles J. U., KC G., Baruah T., Zope R. R. (2017). Chem. Phys. Lett..

[cit38] Korona T., Dodziuk H. (2011). J. Chem. Theory Comput..

[cit39] Williams C. I., Whitehead M. A., Pang L. (1993). J. Phys. Chem..

[cit40] Bucher D. (2012). Chem. Phys. Lett..

[cit41] Farimani A. B., Wu Y., Aluru N. R. (2013). Phys. Chem. Chem. Phys..

[cit42] Hashikawa Y., Murata M., Wakamiya A., Murata Y. (2016). Angew. Chem., Int. Ed..

[cit43] Hashikawa Y., Murata M., Wakamiya A., Murata Y. (2017). J. Org. Chem..

[cit44] Hashikawa Y., Murata Y. (2017). J. Am. Chem. Soc..

[cit45] Huang D. L., Dau P. D., Liu H. T., Wang L. S. (2014). J. Chem. Phys..

[cit46] Zhu G. Z., Hashikawa Y., Liu Y., Zhang Q. F., Murata Y., Wang L. S. (2017). J. Phys. Chem. Lett..

[cit47] Wang L. S. (2015). J. Chem. Phys..

[cit48] Wang X. B., Wang L. S. (2008). Rev. Sci. Instrum..

[cit49] León I., Yang Z., Liu H. T., Wang L. S. (2014). Rev. Sci. Instrum..

[cit50] Broggi J., Terme T., Vanelle P. (2014). Angew. Chem., Int. Ed..

[cit51] Mahesh M., Murphy J. A., LeStrat F., Wessel H. P. (2009). Beilstein J. Org. Chem..

[cit52] Garcia G. A., Nahon L., Powis I. (2004). Rev. Sci. Instrum..

[cit53] Dribinski V., Ossadtchi A., Mandelshtam V. A., Reisler H. (2002). Rev. Sci. Instrum..

[cit54] Wang L. S., Ding C. F., Wang X. B., Nicholas J. B. (1998). Phys. Rev. Lett..

[cit55] Wang L. S., Wang X. B. (2000). J. Phys. Chem. A.

[cit56] Parker S. F., Bennington S. M., Taylor J. W., Herman H., Silverwood I., Albers P., Refson K. (2011). Phys. Chem. Chem. Phys..

[cit57] Wigner E. P. (1948). Phys. Rev..

[cit58] Frisch M. J., Trucks G. W., Schlegel H. B., Scuseria G. E., Robb M. A., Cheeseman J. R., Scalmani G., Barone V., Mennucci B., Petersson G. A., Nakatsuji H., Caricato M., Li X., Hratchian H. P., Izmaylov A. F., Bloino J., Zheng G., Sonnenberg J. L., Hada M., Ehara M., Toyota K., Fukuda R., Hasegawa J., Ishida M., Nakajima T., Honda Y., Kitao O., Nakai H., Vreven T., Montgomery Jr J. A., Peralta J. E., Ogliaro F., Bearpark M., Heyd J. J., Brothers E., Kudin K. N., Staroverov V. N., Kobayashi R., Normand J., Raghavachari K., Rendell A., Burant J. C., Iyengar S. S., Tomasi J., Cossi M., Rega N., Millam J. M., Klene M., Knox J. E. J., Cross B., Bakken V., Adamo C., Jaramillo J., Gomperts R., Stratmann R. E., Yazyev O., Austin A. J., Cammi R., Pomelli C., Ochterski J. W., Martin R. L., Morokuma K., Zakrzewski V. G., Voth G. A., Salvador P., Dannenberg J. J., Dapprich S., Daniels A. D., Farkas Ö., Foresman J. B., Ortiz J. V., Cioslowski J. and Fox D. J., Gaussian 09, Revision C.01, Gaussian, Inc., Wallingford CT, 2010.

[cit59] Ning C. G., Hajgato B., Huang Y. R., Zhang S. F., Liu K., Luo Z. H., Knippenberg S., Deng J. K., Deleuze M. S. (2008). Chem. Phys..

